# Cognitive-Behavioral and Emotion-Focused Couple Therapy: Similarities and Differences

**DOI:** 10.32872/cpe.v2i3.2741

**Published:** 2020-09-30

**Authors:** Guy Bodenmann, Mirjam Kessler, Rebekka Kuhn, Lauren Hocker, Ashley K. Randall

**Affiliations:** a Clinical Psychology for Children/Adolescents and Couples/Families, University of Zurich, Zurich, Switzerland; b Counseling and Counseling Psychology, Arizona State University, Tempe, AZ, USA; Philipps-University of Marburg, Marburg, Germany

**Keywords:** couple therapy, cognitive behavioral couple therapy, emotion-focused couple therapy, efficacy

## Abstract

**Background:**

Couples and families often seek therapy to deal with relational distress, which is a result of external or internal factors of the relationship. Two approaches are acknowledged to be most effective in dealing with relationship distress or psychological disorders in couples: (a) cognitive behavioral couple therapy with new directions (CBCT) and (b) emotion-focused couple therapy (EFCT). In this article we investigate how much CBCT and EFCT really differ with regard to working with emotions, which is claimed to be a major focus of EFCT, and whether there exist significant differences in efficacy between these two approaches.

**Method:**

This article critically reviews the theoretical background, process, techniques and outcomes associated with CBCT and EFCT in an effort to challenge the assumptions noted above.

**Results:**

There is no evidence that EFCT is more emotion-focused than CBCT. Both approaches were repeatedly examined with RCT studies with follow-ups. In sum, no significant differences in effect size were found between CBCT and EFCT.

**Conclusion:**

CBCT and EFCT are both effective in reducing couples’ distress.

## Abstract (highlights)

- CBCT and EFCT are both effective in helping couples deal with relationship distress.
- Both are similarly effective in helping couples to better understand and cope with their presenting concerns.
- Both approaches address the importance of personal schema, triggering relevant cognitions and emotions.
- Both approaches help couples wherein one partner has been diagnosed with a clinical disorder.

For couples seeking couple therapy, there is broad international empirical evidence advocating that couple therapy is advantageous in reducing relationship distress and improving relationship quality. Overall, couple therapy exhibits excellent efficacy with an internationally established mean effect size of *d* = 0.95, ranging from *d* = 0.59 to 1.03 (e.g., Shadish & Baldwin, 2003, 2005).

Among a large range of different therapeutic approaches, cognitive-behavioral couple therapy (CBCT) and emotion-focused couple therapy (EFCT) are amongst the most widely applied couples’ interventions. CBCT as well as EFCT have repeatedly been examined regarding their efficacy. Some claim that EFCT outperforms CBCT and represents the most effective approach for treating relationship problems (e.g., Roesler, 2018). However, an ancient meta-analysis revealed only marginal differences between the various approaches (Shadish & Baldwin, 2005). The purpose of this review is to analyze recent studies on efficacy of both approaches and to test the assumption that EFCT (attachment based) is more emotion-focused than CBCT (learning based).

## Brief Review of the Theoretical Underpinnings of CBCT and EFCT

In this section, we will provide a brief overview of the theoretical underpinnings and common methods used in CBCT and EFCT. Denominations of *emotion-focused* versus *cognition-focused* are tested regarding their meaning for clinical work.

### Cognitive-Behavioral Couple Therapy

#### Background

Cognitive-behavioral couple therapy (CBCT) relies on principles from social learning theories and focuses on the interplay between partners’ cognitions, behaviors, and emotional responses to help them improve their communication and problem-solving (Epstein & Zheng, 2017). CBCT draws on concepts stemming from behavioral couple therapy, cognitive therapy, as well as empirical findings in basic research (Baucom et al., 2008). Therapists working from a CBCT lens aim to improve partners’ skills (e.g., communication and problem-solving skills), modify dysfunctional cognitions and attitudes, in an attempt to improve relationship quality and decrease emotional distress such as anger, sadness or disgust (Epstein & Baucom, 2002; Epstein & Zheng, 2017).

#### Process

The goal of CBCT is to help partners restructure cognitions that may yield relational distress, which include unrealistic expectations, dysfunctional attributions and irrational assumptions (Epstein & Zheng, 2017). CBCT operates under the premise that cognitions cause emotions and subsequent behaviors (e.g., the *cognition* “you do not care about me” may lead to *emotions* such as anger and sadness that motivates coercive *behavior* to get more attention). Thus, the assumption of CBCT is that negative mood (dissatisfaction) and emotions (anger, disappointment, frustration, resignation), reflected in deleterious behaviors (i.e., generalized criticism, defensiveness, belligerence, contempt, aggression or violence), are a major motive why couples seek for interventions (Bradbury & Bodenmann, 2020).

#### Techniques

One of the common techniques used in CBCT is cognitive restructuring, wherein the clinician guides partners to “identify and evaluate cognitions as they occur” (Epstein & Zheng, 2017, p. 143). Dysfunctional cognitions, either regarding irrational beliefs, dysfunctional expectancies or negative attribution styles are viewed as the causes of negative emotions (Bradbury & Fincham, 1990). CBCT aims to strengthen partners’ communication skills in order to allow partners to safely disclose their needs and emotions, without risk of their partner’s negative reactions. Therefore, instead of blaming the partner, partners learn to express their sentiments and needs using speaker-listener rules and techniques. CBCT also applies *cognitive-emotional techniques* such as cognitive restructuring (i.e., identifying and disputing irrational thoughts leading to negative emotions) (e.g., Baucom et al., 2019).

More recent approaches such as the *integrative behavioral couple therapy* (IBCT; Jacobson & Christensen, 1996) and *coping-oriented couple therapy* (COCT; Bodenmann, 2010) also refer to CBCT principles. However, IBCT focuses on acceptance in addition to the above-mentioned techniques and tries to improve couples’ mutual tolerance. COCT focuses on stress and its impact on couples’ functioning. This approach addresses mutual emotional understanding facing stress-related negative behaviors towards the partner. By means of the 3-phase-method, partners learn to engage in deepened emotional self-disclosure, empathic listening and providing emotion-focused support (i.e., dyadic coping) that matches the partners’ needs. By doing this, emotional bonding, mutual intimacy and closeness as well as mutual trust between partners are enhanced (Bodenmann & Randall, 2020). In sum, techniques used in CBCT aim at improving partners’ skills in an attempt to modify dysfunctional cognitions, emotions and behaviors or to accept them under specific circumstances.

#### Outcomes

CBCT has shown to be effective in improving couples’ function. In addition, positive effects are reported regarding partner’s psychological (e.g., PTSD and OCD) and physical health (e.g. cancer), as well as other severe stressors that may yield relational concerns (for a review see Epstein & Zheng, 2017).

### Emotion-Focused Couple Therapy

#### Background

Emotionally focused couple therapy (EFCT) is an experiential, humanistic and systemic therapy grounded in attachment theory and social neuroscience (Greenman, Johnson, & Wiebe, 2019). EFCT does not directly focus on skill training, rather, the focus is to build new emotional experiences between partners that foster attachment security (Wiebe & Johnson, 2016). The original framework of EFCT proposed that distress in the relationship could be repaired though regulation of emotions by the other partner (Greenberg & Johnson, 1988). This was later adapted to include foundations of attachment theory as well as working to increase both partner’s emotional self-regulation and other regulation (Greenberg & Goldman, 2008; Johnson, 2004). EFCT primarily aims to facilitate the expression of primary emotions (such as feelings of hurt, feelings of inadequacy and deprivation of love, respect and appreciation) and to understand these feelings behind secondary emotions such as anger or contempt (Greenberg & Johnson, 1988).

#### Process

The overarching goals of EFCT is to have partners access and reprocess their emotional experiences to restructure partners’ interaction patterns. The outcome of this approach is to help partners learn new aspects about themselves and develop a more functional pattern of interaction with their partner that is matching with their specific attachment needs (Johnson, 2019). Within EFCT, the therapist tries to strengthen the attachment bond between partners by addressing the intrapsychic (attachment-related experiences) and interpersonal perspective regarding dysfunctional interaction patterns of distressed partners. Emotion-focused couple therapy understands these patterns as the result of an insecure attachment bond where both partners signal attachment distress in a way that inadvertently keeps their partner at a distance (Greenman et al., 2019).

Typically, EFCT is differentiated in three stages (Greenman et al., 2019). In the first stage (*cycle de-escalation*), the therapist tracks and reflects the pattern of interaction with the couple and tries to identify negative patterns wherein the partners may “criticize/attack” one another, which is often followed by “defensiveness/distance”. These interaction patterns are viewed as hindering constructive emotional exchange. The goal of the first stage is to gain a meta-perspective of the couples’ interaction by realizing that the partners’ dysfunctional interaction maintains both partners’ attachment insecurity and causes emotional distress. In the second stage (*restructuring interactions*), the therapist tries to give insight into new emotional experiences by facilitating new interactions, which will help lead to secure bonding. The therapist helps to explore attachment vulnerabilities that partners share with each other. In this method, partners learn how to respond to the other in an emotionally attuned and supportive way. Instead of blaming or withdrawing from the partner, partners learn to become more responsive to the other; increasing their awareness of their partner’s attachment needs. Instead of negativity, primary emotions such as sadness, fear or shame are expressed. The therapist helps the speaker to find adequate wording for their emotional state. In the third stage (*consolidation*), partners learn new ways of solving problems that become possible based on their secure attachment experience.

#### Techniques

A primary focus in EFCT is helping couples learn how to communicate their emotions more effectively with one another (Gladding, 2015). Couples are instructed to better perceive their emotions and to engage more in mutual responsiveness and dyadic engagement (Burgess Moser & Johnson, 2008). Hence, in EFCT, couples are encouraged to explore here-and-now emotional experiencing (Greenman et al., 2019). Instead of sharing primary emotions, distressed couples often communicate secondary emotions expressed in attacking, nagging, and withdrawing. As such, the EFCT therapists help guide each partner to uncover primary emotions (sadness, fear, shame, etc.). The therapist guides both partners, working out primary emotions for one, and showing the other partner how to listen emotionally engaged and how to respond in an emotionally attuned way. The “new emotional music then elicits new responses and, gradually, changes the dance between partners” which means that new behavioral interaction patterns can be established (Wiebe & Johnson, 2016, p. 390).

Common techniques within EFCT include *bonding* and *enactments*. Therapists guide couples through the conversations about emotion and encourage each partner to engage in a release of that emotion, to increase self-awareness (Gladding, 2015). This process leads to the therapeutic technique of *bonding*, which is when the partner who is hearing the emotional response can become more aware of their partner’s perception, thus increasing empathy. *Enactments*, reminiscent of Gestalt therapy, help each partner explore and express deeper emotions by engaging in role-play or two-chair techniques (Gladding, 2015).

#### Outcomes

Various studies have shown EFCT’s effectiveness with couples in distress, couples coping with post-traumatic stress disorder (PTSD), and couples coping with chronic illness (Bailey, 2002; Beckerman, 2004; Bradley & Johnson, 2005). Additionally, EFCT has been effective in increasing intimacy between partners (Soltani et al., 2013).

### Similarities and Differences Between Both Approaches

CBCT and EFCT approaches are grounded in different theories and, as such have a different conceptualization of the development and maintenance of relationship distress. Traditional CBCT is skill-oriented and aimed at teaching couples’ new ways of communication and conflict resolution. Methods are a highly structured and often manualized, such as the communication training. New directions in CBCT, like the acceptance approach (Jacobson & Christensen, 1996) or 3-phase-method (Bodenmann, 2010) further expand these methods by focusing on insight-oriented empathic understanding and deepened emotional experiences in the case of the latter approach. All techniques in CBCT, however, focus on the interplay between cognitions and emotions as the major outcome of interest. However, instead of working directly with emotions, therapists address dysfunctional thinking and information processing, negative and unrealistic or exaggerated attitudes towards the partner and their impact on couple’s emotional experiences and behaviors. Thus, techniques utilized in CBCT focus on modifying cognitive distortions with the goal to tap into the emotional exchange between partners. COCT and IBCT further offer techniques directly allowing shared emotional experiences like this is the case in the 3-phase-method or the empathic joining technique.

EFCT is considered an experiential approach that enables partners to develop new feelings and interaction patterns. It primarily focuses on attachment schemas or personal needs of belonging, being respected and validated. Partners learn to understand that negative emotions and dysfunctional interaction patterns result from the non-fulfilment of these attachment needs. Instead of a structured training like in CBCT, the EFCT-therapists work with emotional experiences during partners’ interactions by making them visible and tangible.

Creating emotional and cognitive awareness of the partner’s insecure attachment is a key component of this approach. EFCT-therapists explain emotional reactions and search together with the partners for an attachment-based understanding. Thus, the goals are somewhat similar in EFCT and CBCT (compare 3-phase-method), however, the methods vary. EFCT-therapists are not teaching skills, their approach is less structured and therapists are more active in uncovering processes. CBCT-therapists are similarly allowing emotional experiences and emotional understanding, but by using techniques such as Socratic questioning or the method of prompting (therapists explore and reinforce relevant cognitions and deeper emotions, ask open-ended questions and guide smoothly to the personally relevant construct that may be an attachment scheme, but can also be any other type of schema).

In sum, both CBCT and EFCT approaches aim to address relationship distress, with the goal of helping couples deal more effectively with negative emotions. Both approaches work with partners’ emotional experience, however, the ways in which each method addresses them is different.

### Efficacy of CBCT and EFCT

In psychotherapy research, minimal differences in outcomes of the various approaches are reported (Wampold et al., 2002). While some psychotherapies show higher effectiveness in treating specific disorders (e.g., CBCT for anxiety disorders), in general, common factors such as the therapeutic alliance account for more variance than specific treatment modality. Correspondingly, Wampold et al. (2002) report that only 1% of the variability of treatment outcome can be explained by a specific treatment.

Findings are similar in couple therapy and again, differences between various approaches are minimal (Christensen & Heavey, 1999).

#### Efficacy of CBCT

CBCT is considered one of the most widely evaluated therapeutic approaches for working with couples. Since the 1980ies, several dozens of RCT-studies have supported the effectiveness and efficacy of CBCT (Bradbury & Bodenmann, 2020). 70% of the couples improved after CBCT (Baucom et al., 1998), and 50% show stable effects over a period of five years (Christensen et al., 2010). Christensen et al. (2004) reported 71% of clinical recovery in integrated CBCT compared to 54% in classical CBCT. According to this study, CBCT proves to be efficient in the long term, with an effect size of *d* = 0.92 at the 5-year follow-up, slightly outperformed by ICBT (*d* = 1.03) (Christensen & Glynn, 2019). Bodenmann et al. (2008) reported effect sizes of *d* = 1.46 at the 6-months follow-up and *d* = 1.74 at the one-year follow-up of coping-oriented CBCT in treating depression. In the various meta-analysis, effect sizes for CBCT ranged from *d* = 0.53 (Rathgeber et al., 2019) up to *d* = 0.95 (Byrne et al., 2004).

#### Efficacy of EFCT

The efficacy of EFCT has been examined in 10 RCT-studies, all which support its efficacy. However, these studies do not always present classical effect sizes. In the meta-analysis by Johnson et al. (1999), including four randomized trials, an effect size of *d* = 1.31 is reported. More recently, Beasley and Ager (2019) published a new meta-analysis that included studies that were conducted and published since the last meta-analysis, covering a period of 19 years. In this meta-analysis, nine RCT studies were included. However, authors did not calculate Cohen’s *d*, but Hedges’s *g*. Thus, results are not directly comparable with previous research or studies related to CBCT. Hedges’s *g* was 2.09 (Beasley & Ager, 2019).

In earlier research on EFCT, Johnson and Talitman (1997) report an improvement in relationship quality in 50% of couples (no RCT-study) at post-test, while 70% showed recovery at 3-month follow-up. In a recent study (Wiebe et al., 2017), 61% fully recovered, 11% improved (but no recovery), 25% remained unchanged and 4% showed a deterioration.

#### Comparison of Intervention Studies and Meta-Analyses

Statements on the efficacy of CBCT are based on a great number of studies (*N* = 86 studies in the different meta-analyses), usually relatively large samples, and randomized controlled trials (which represent the Golden standard in treatment evaluation studies). The evaluation of EFCT is based on fewer studies (*N* = 32 studies in the different meta-analyses), not always RCT designs and usually smaller samples. The above-cited most recent meta-analysis by Beasley and Ager (2019) on the effectiveness of EFCT included only four methodologically sound RCT studies, and the first meta-analysis (Johnson et al., 1999) had also included only four trials. Only 0.01% of all conducted evaluation studies in EFCT could be included in this latest meta-analysis because of insufficient methods or sample sizes or other statistical shortfalls. Thus, only four follow-up studies out of nine met inclusion criteria within the last 19 years (Beasley & Ager, 2019). The mean sample size in these studies was considerably small with *N*_mean_ = 14 in the intervention group versus *N*_mean_ = 13 in the intervention group. Three out of nine studies were rated to not meet criteria for treatment integrity and the others were at least acceptable (Beasley & Ager, 2019). Often studies were not in the context of relationship distress but related to other problems such as medical issues (e.g., infertility, end-stage cancer or psychological disorders such social anxiety, depression). They represented no “pure” studies on effects of EFCT on relationship distress.

More interesting than reviews and meta-analyses on one single approach are studies directly comparing both approaches. The meta-analysis with 33 suitable primary studies by Rathgeber et al. (2019) is such an example (*n* = 21 studies on CBCT, *n* = 12 studies on EFCT). In this study, a total of 2,730 participants were included. Results reveal a medium overall effect size at post-test *g* = 0.60 (Behavioral cognitive therapy (BCT): *g* = 0.53; EFCT: *g* = 0.73). After 6 months smaller effects were reported (overall: *g* = 0.44; BCT: *g* = 0.35; EFCT: *g* = 0.66). Most important, no significant differences in effect sizes were found between the two couple therapy approaches. This finding echoes results of the study by Byrne et al. (2004), where large effect sizes for both treatments (*d*_BCT_ = 0.95, *d*_EFCT_ = 1.27) on quality of couples’ relationships compared to waiting-list controls are reported. “Taken together, meta-analyses of existing efficacy studies continue to support an approximate *d* of at least 0.80 for BCT and EFCT, with 60–72% of couples experiencing reliable pre–post improvements in satisfaction” (Bradbury & Bodenmann, 2020, p. 102).

## Conclusion

In sum, CBCT and EFCT are both effective in helping couples deal with relationship distress (Bradbury & Bodenmann, 2020). Based on our review of the literature, it is important to acknowledge that while both approaches have their strengths and weaknesses, both are similarly effective in helping couples to better understand and cope with their presenting concerns. Additionally, both approaches address the importance of personal schema, triggering relevant cognitions and emotions. The assumption that CBCT is purely behavioral, focusing on cognitions and neglecting emotions is often wrongly derived from the designation, but lacks any theoretical and practical basis. CBCT and EFCT both address similarly the emotional experiences between partners; however, each approach does so differently. Both approaches have been found to be beneficial in improving relationship distress and helping couples overcome their relational difficulties, in addition to helping couples wherein one partner has been diagnosed with a clinical disorder. It is important that clinicians and policy makers are aware of these two evidence-based approaches, and expand their application to other areas wherein couples may be experiencing distress (e.g., health psychology). Therefore, publications building public awareness for the use of couple therapy in treating psychological disorders are important (Fischer et al., 2016; Leuchtmann & Bodenmann, 2017).
